# α2-fraction and haptoglobin as biomarkers for disease activity in oligo- and polyarticular juvenile idiopathic arthritis

**DOI:** 10.1186/s12969-022-00721-7

**Published:** 2022-08-13

**Authors:** Ludwig Zeller, Pascal N. Tyrrell, Stella Wang, Nadine Fischer, Johannes-Peter Haas, Boris Hügle

**Affiliations:** 1 German Centre for Pediatric and Adolescent Rheumatology (GCPAR), Gehfeldstrasse 24, 82467 Garmisch-Partenkirchen, Germany; 2 Department of Medical Imaging, University of Toronto, Toronto, Canada; 3 Department of Statistical Sciences, Institute of Medical Science, University of Toronto, Toronto, Canada

## Abstract

**Objectives:**

Unlike in adult rheumatology, for most forms of juvenile idiopathic arthritis (JIA) no reliable biomarkers currently exist to assess joint and disease activity. However, electrophoresis is frequently found changed in active juvenile arthritis. The objective of this study was to evaluate the α2-fraction of serum electrophoresis and its main components as biomarkers for JIA, categories extended/persistent oligoarthritis and seronegative polyarthritis, in comparison with the conventionally used erythrocyte sedimentation rate and C-reactive protein.

**Methods:**

Serum samples and clinical data from 181 patients with JIA were collected. Serum electrophoresis and α2-fraction and its components were determined using standard methods. Relationship between calculated α2-fraction of serum electrophoresis (CA2F) and its components, acute-phase parameters and cJADAS27 was assessed using Pearson’s correlation coefficient and linear regression modelling, adjusting for confounding effects. Results were confirmed in a second cohort with 223 serum samples from 37 patients, using a mixed model to account for repeated measures.

**Results:**

Compared to ESR and CRP, CA2F showed higher correlation to cJADAS27, in particular for persistent oligoarthritis. Of the three components of the α2-fraction, haptoglobin showed the highest correlation to cJADAS27. Regression analysis demonstrated higher ability to predict cJADAS27 for CA2F, and especially for haptoglobin as a component thereof, than for CRP and ESR.

**Conclusion:**

Compared to conventional methods, α2-fraction of serum electrophoresis and specifically, haptoglobin show higher correlations with disease activity in common subtypes of JIA, representing excellent candidates as biomarkers for disease activity. Further studies are necessary to determine diagnostic value and correlations in other subtypes.

**Supplementary Information:**

The online version contains supplementary material available at 10.1186/s12969-022-00721-7.

## Introduction

Juvenile idiopathic arthritis (JIA) is the most common rheumatic disease in childhood, affecting up to 4 per 1,000 children, and is characterized by chronic inflammation of the joints in children and adolescents [1]. JIA is not a single disease entity, but encompasses all forms of arthritis that begin before age 16 and persist for more than 6 weeks and is of unknown origin, divided into seven categories [2]. The pathology of JIA is believed to be of complex, multifactorial origin, with both genetic and environmental factors influencing disease origin and outcome [3, 4].

Conventional biomarkers for rheumatic diseases, especially C-reactive protein (CRP) and erythrocyte sedimentation rate (ESR), have shown excellent correlation to disease activity in adult diseases such as rheumatoid arthritis and ankylosing spondylitis and are regularly used in clinical practice [5]. In pediatric rheumatology, there are no similar biomarkers for JIA available to practitioners: most of the categories lack reliable biomarkers for disease activity beyond musculoskeletal ultrasound, except for a small number of JIA subsets, namely systemic JIA and to a lesser extent, rheumatoid-factor positive JIA and juvenile spondyloarthritis [6, 7]. While in fact the proposed disease index for JIA (Juvenile Arthritis Disease Activity Score, JADAS) included ESR as an activity marker, newer versions of the index (clinical JADAS, or cJADAS) use clinical markers alone [8–10]. The relative value of ESR and CRP in evaluation of JIA is doubtful, and clinical practitioners usually rely much more on clinical examination and other techniques such as ultrasound to assess disease activity [4].

It has been observed by the authors that in serum electrophoresis, an established method to examine blood globulins, the α2-fraction as a biomarker of acute-phase reaction is frequently elevated in JIA patients with active disease, especially in patients with oligoarthritis. The purpose of this study was to evaluate the use of the calculated α2-fraction of serum electrophoresis (CA2F) and its components as biomarkers of disease activity in JIA patients. The objectives were: 1) to describe the relationship between ESR, CRP, CA2F and its components, and cJADAS27 as index for disease activity in JIA, and 2) to determine if the correlation between predictors of cJADAS27 differ by categories of oligoarthritis (persistent and extended) and rheumatoid-factor negative polyarthritis. These categories were chosen primarily as they represent the majority of cases of JIA, and as CRP and ESR possibly play a more prominent role as biomarkers in other categories.

## Patients and methods

### Patients

An exploratory cohort (ExpC) was formed, consisting of 181 single serum samples, from patients with a confirmed diagnosis of oligoarticular (extended and persistent) JIA and polyarticular JIA according to ILAR (International League of Associations for Rheumatology) criteria, collected at the biobank of the German Centre for Paediatric and Adolescent Rheumatology.

Subsequently, a second confirmatory cohort (ConfC) was established maximizing sample numbers from single patients. This cohort consisted of 223 serum samples from 37 patients, with a median of 6 samples per patient (range 3 to 12). 59 samples had already been included in the exploratory cohort as single serum samples, bringing the number of total samples investigated to 345 samples. A diagnosis of JIA was confirmed by the admitting physician and the presence of diseases possibly affecting haptoglobin serum levels was excluded [11, 12]. Patients and caregivers gave informed consent prior to inclusion into the biobank. The following data was extracted from patient files: age at diagnosis of JIA, gender, age at performance of the test, JIA category, presence of antinuclear antibodies, total active joint count, and the cJADAS27 as a composite marker for disease activity. The cJADAS27 was chosen instead of JADAS27 for its exclusion of ESR as a component parameter [8, 13]. The study was approved by the Ethics Committee of the Medical Faculty, Ludwig-Maximilians University Munich, Germany (Nr. 11,073) and was performed in accordance with the Declaration of Helsinki.

### Serum analysis

ESR (normal range: newborn to puberty 3 to 13 mm/h, males < 15 mm/h, females < 20 mm/h) was analyzed using Microvette CB 200 tubes (Sarstedt, Germany). Serum electrophoresis was performed using a Capillarys 3 Tera electrophoresis system (Sebia, Lisses, France). Total serum protein (normal range: children 5.7 – 8.0 g/dl, adults 6,4—8,3), C-reactive protein (normal range: < 0.5 mg/dl), α2-macroglobulin (normal range: 130—300 mg/dl) and haptoglobin (normal range: 30—200 mg/dl) were determined using a cobas 8000 modular analyzer (Roche, Grenzach-Wyhlen, Germany) and ceruloplasmin (normal range: children 23–43 mg/dl, adults 20–60 mg/dl) was measured using a BN II system (Siemens, Erlangen, Germany). The calculated absolute α2-fraction (CA2F) was computed as the product of the total serum protein (in g/dl) and the α2-fraction (in %) to obtain an estimate of the absolute amount of protein present in the α2-fraction.

### Statistics

Demographic data were analyzed using descriptive statistics. cJADAS27 was transformed through a natural logarithm after adding 1 to normalize residuals without losing sample size. Pearson’s correlation coefficients were used for pairwise comparisons of serum analytes.

Multivariable linear regression models and linear mixed effect models were built for ESR, CRP, CA2F and haptoglobin separately for ExpC and ConfC, respectively, both adjusting age at diagnosis, and category of diagnosis as covariates. Gender, age at testing and antinuclear antibodies were removed from the model due to multicollinearity and insignificant confounding effect. For the ConfC, a linear mixed effect model was used to account for repeated measures by introducing random effect. Time and the interaction term between time and the main predictor was adjusted in the models to assess longitudinal effect.

R squared was calculated to assess the proportion of variability explained by each predictor itself for the ExpC model. F statistics and the p-values were reported by each predictor from each model. Multivariable linear regression was also modelled using only the first sample per patient for the ConfC as a sensitivity analysis.

Mean levels and standard deviation of haptoglobin, CA2F, ESR and CRP were calculated for low, median and high disease activity according to the definitions of the cJADAS27 [14]. Area under the curve (AUC) and optimal cut-off values were determined for low disease activity definition of the cJADAS27 using receiver operating characteristic (ROC) analysis. In the ROC analysis, Youden’s J statistic was calculated to determine the optimum of sensitivity and specificity [15].

SAS version 9.4 for Windows and SPSS version 26 for Windows were used for the analysis.

## Results

The demographic data of the patients is shown in Table 1. Two outliers were identified in the ExpC, but removal in the analyses did not change the results significantly so they were included in the subsequent analysis.

**Table 1:** Patient characteristics of investigated cases

Variables	Exploratory Cohort (ExpC)	Confirmatory Cohort (ConfC)
Female, no (%)	143/181 patients (79.0%)	27/37 patients (73.0%)
Age at diagnosis (median, range)	4.8 years (1.1 -16.3)	4.61 years (1.06 -15.47)
Antinuclear antibodies	134/181 patients (74.0%)	22/37 patients (59.4%)
Disease duration at time of testing	14 months (0–170)	n/a
Diagnosis		
oligoarthritis, persistent	75/181 (41.4%)	9/37 (24.3%)
oligoarthritis, extended	31/181 (17.2%)	10/37 (27.0%)
rheumatoid factor negative polyarthritis	75/181 (41.4%)	18/37 (48.6%)
Total active joint count (TAJC) (mean, standard deviation)		
oligoarthritis, persistent	1.17 ± 1,66^a^	0.86 ± 1,17^a^
oligoarthritis, extended	1.98 ± 3.41^a^	1,44 ± 1.88^a^
rheumatoid factor negative polyarthritis	4.53 ± 7.65^a^	3.09 ± 7.22^a^
cJADAS27 (mean, standard deviation)		
oligoarthritis, persistent	0.93 ± 1.19^a^	3.47 ± 4.02^a^
oligoarthritis, extended	1.50 ± 2.60^a^	5,37 ± 4.81^a^
rheumatoid factor negative polyarthritis	2.79 ± 4.35^a^	5.42 ± 7.01^a^

^a^ Data counted per sample

### α2-Fraction of Electrophoresis shows higher correlation to joint activity in persistent and extended oligoarthritis

In the ExpC, ESR (normal range) and CRP (normal range) correlated modestly with joint activity as measured by the cJADAS27 (*r* = 0.248, *p* < 0.001 and *r* = 0.243, *p* < 0.001, respectively for the whole sample) (Supplemental Table 1). α2-fraction of electrophoresis (normal range) (*r* = 0.202, *p* < 0.001) and CA2F (*r* = 0.233, *p* < 0.001) yielded similar values for the whole sample, with CA2F showing a slightly higher correlation. For total oligoarthritis and especially, persistent oligoarthritis, CA2F showed higher correlation to the cJADAS27 (*r* = 0.340, *p* < 0.001 and *r *= 0.398, *p* < 0.001) than ESR (*r* = 0.321, *p* < 0.001 and *r* = 0.311, *p* = 0.001) and CRP (*r* = 0.244, *p* = 0.002 and *r* = 0.200, *p* = 0.033).

### Haptoglobin as a component of the α2-fraction shows a higher correlation to cJADAS27 than the α2-fraction as a whole

The three major components of the α2-fraction (α2-macroglobulin, haptoglobin and ceruloplasmin) were determined for each sample in the ExpC, and correlation to cJADAS27 was calculated (Supplemental Table 1). While α2-macroglobulin showed minimal correlation to cJADAS27, ceruloplasmin showed a correlation profile similar to ESR, with the largest correlation in persistent oligoarthritis (*r* = 0.405, *p* < 0.001). Haptoglobin showed in general the highest correlation to cJADAS27, with *r* = 0.339, *p* < 0.001 overall, *r* = 0.415, *p* < 0.001 for polyarthritis, and *r *= 0.472, *p* < 0.001 for persistent oligoarthritis. Differences in the correlations in the various categories were partly explained by the patients with extended oligoarthritis having a higher disease duration (5.2 ± 3.7 years) than both persistent oligoarthritis (2,1 ± 3,4 years) and seronegative polyarthritis (2.2 ± 2.6 years).

In the ConfC, these results were confirmed, with haptoglobin showing the highest correlation to cJADAS27 (*r* = 0.440, *p* < 0.001), compared to CRP (*r* = 0.292, *p* < 0.001) and ESR (*r* = 0.420, *p* < 0.001) in the whole sample (Supplemental Table 2). For the total cohort (ExpC + ConfC combined), correlation was also tested for total active joint count, with lower correlation overall, but again haptoglobin showed the highest correlation (*r* = 0.189, *p* < 0.001), compared to CRP (*r* = 0.165, *p* < 0.001) and ESR (*r* = 0.141, *p* < 0.001) (Supplemental Table 3).

**Table 2:** Logistic regression analysis for cJADAS, and CRP, ESR, CA2F and haptoglobin as single parameters

	Exploratory Cohort (ExpC)				Confirmatory Cohort (ConfC)	
Analyte	Parameter estimate ± standard error	F-value from test of fixed effect (*p* value)	R2 of the predictor	R2 of the model	Parameter estimate ± standard error	F-value from test of fixed effect (*p* value)
CRP (mg/dl)	0.104 ± 0.040	6.8 (0.0099)	0.005	0.067	0.080 ± 0.027	8.48 (0.0042)
ESR (mm/h)	0.008 ± 0.001	17.66 (< .0001)	0.012	0.120	0.015 ± 0.003	22.78 (< .0001)
CA2F (g/l)	1.123 ± 0.264	18.75 (< .0001)	0.012	0.122	1.989 ± 0.405	24.61 (< .0001)
Haptoglobin (mg/dl)	0.003 ± 0.000	33.75 (< .0001)	0.022	0.187	0.004 ± 0.000	39.45 (< .0001)

**Table 3:** Median levels and standard deviation for low, median and high disease activity of cJADAS for CRP, ESR, CA2F and haptoglobin as single parameters

	Haptoglobin	CA2F	ESR	CRP
HDA	180.2 ± 117.3 mg/dl	0.92 ± 0.22 g/dl	28.9 ± 28.5 mm/h	0.88 ± 2.24 mg/dl
MDA	118.2 ± 85.3 mg/dl	0.84 ± 0.19 g/dl	18.0 ± 18.5 mm/h	0.55 ± 2.06 mg/dl
LDA	79.9 ± 56.1 mg/dl	0.75 ± 0.13 g/dl	12.8 ± 13.8 mm/h	0.14 ± 0.30 mg/dl
Total	131.75 ± 101.25 mg/dl	0.85 ± 0.20 g/dl	20.77 ± 22.80 mm/h	0.57 ± 1.88 mg/dl

### Prediction of joint activity by haptoglobin and CA2F is superior to CRP and ESR

Using multivariable linear regression, models were constructed to assess the association between cJADAS27 and all four parameters (ESR, CRP, CA2F and haptoglobin) separately in the ExpC, adjusting for age at diagnosis and disease category as covariates. Regression analysis showed higher F-values for CA2F (F[5,175] = 18.13, *p* < 0.0001) and significantly higher values for haptoglobin (F[5,175] = 33.75, *p* < 0.0001) than for CRP (F[5,175] = 6.80, *p* = 0.0099) and ESR (F[5,175] = 17.66, *p* < 0.0001) (Table 2). The proportion of variability explained by the model using pseudo R squares was 12.2% for CA2F and 18.7% for haptoglobin, compared to 6.6% for CRP and 11.9% for ESR. In single sample multivariable linear regression, effects of each predictor did neither change direction nor significantly change magnitude, but model fit and the accuracy was improved when considering the repeated effect.

In the ConfC, Regression analysis confirmed significantly higher F-values for CA2F (F[1,86] = 24.61, *p* < 0.0001) and haptoglobin (F[1,86] = 39.45, *p* < 0.0001) than for CRP (F[1,85] = 8.48, *p* = 0.0042) and ESR (F[1,86] = 22.78, *p* < 0.0001) (Table 2).

### Determining optimal cut-off levels for haptoglobin and CA2F for low disease activity of cJADAS27

**Fig. 1:**
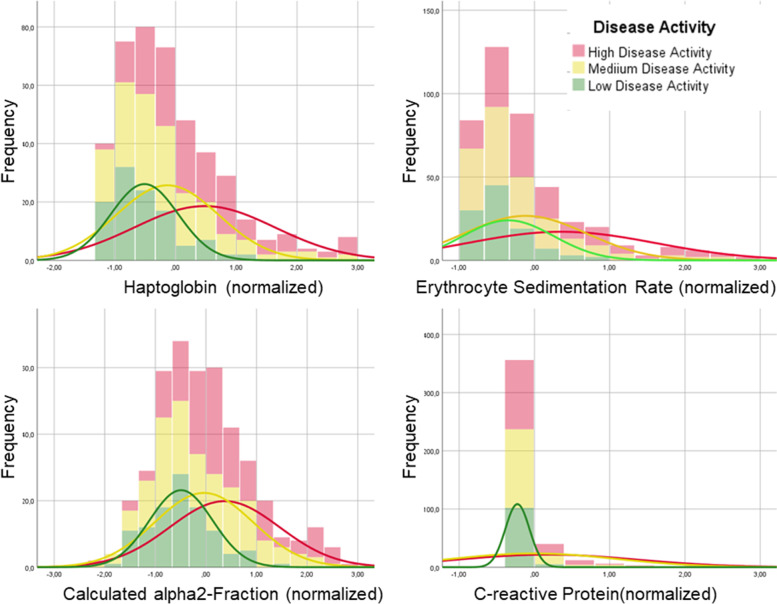
Distribution of normalized values for haptoglobin, calculated alpha2-fraction, ESR and CRP in oligo- and polyarticular JIA combined, according to disease activity measured by cJADAS27 in the combined Cohorts (ExpC + ConfC) with all samples. Mean curves are shown for the three levels (LDA, MDA and HDA)

**Fig. 2:**
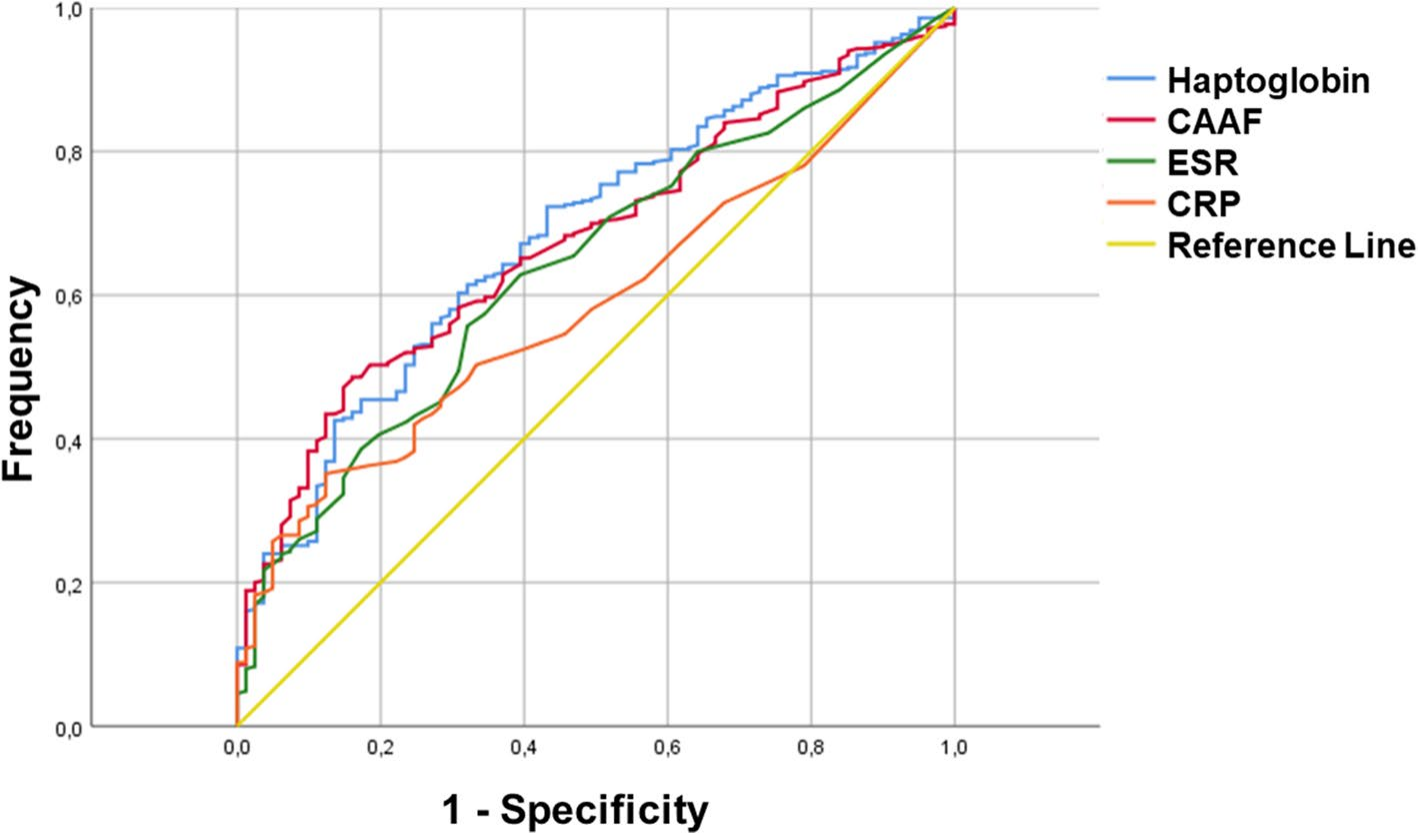
ROC curve for low disease activity measured by cJADAS27 for haptoglobin, CA2F, ESR and CRP in the combined Cohorts (ExpC + ConfC) with all samples

Using the definitions for low or minimal disease activity (LDA), moderate disease activity (MDA) and high disease activity (HDA) of the cJADAS27 for oligo- and polyarticular diseases, mean levels were calculated for the four parameters in the combined Cohorts (ExpC + ConfC) using all available samples (Table 3) (Fig. 1) [14]. Using ROC analysis, AUC was calculated for haptoglobin at 0.725, for CA2F at 0.699, for ESR at 0.622, and for CRP at 0.615. An optimal cut-off for LDA was calculated for haptoglobin at 79.85 mg/dl, with a sensitivity of 75.8% and a specificity of 59.6% (Fig. 2). For CA2F, this was calculated at 0.86 g/dl, with a sensitivity of 50.3% and a specificity of 86.2%. For comparison purposes, for ESR best cut-off was calculated at 13.5 mm/h (sensitivity 58.1%, specificity 68.8%), and for CRP at 0.21 mg/dl (sensitivity 36.6%, specificity 86.2%).

## Discussion

For adult rheumatology practitioners, it is hard to fathom how little of a role laboratory biomarker play in the clinical practice of pediatric rheumatologists. Adult practitioners can use ESR and especially CRP routinely as a fairly reliable marker of disease activity, where both parameters can regularly be expected to correlate with disease activity and fall after initiation of treatment [5, 16, 17]. In RA, both ESR and CRP have been validated in the context of the DAS28 against radiographic progression and physical function [18].

In pediatric rheumatology, laboratory testing in oligo- and polyarticular JIA, which comprise approximately two thirds of all JIA cases, so far plays only a minor role in evaluating disease activity [19]. In textbooks, changes in inflammatory parameters seen in these categories are described as “mild to moderate elevation of the erythrocyte sedimentation rate […] and elevation of C-reactive protein levels may occur” [20]. ESR especially is described as variable in both oligo- and polyarthritis, with further differentiation of polyarthritis into subsets with possibly low or high ESR levels attempted by some authors to explain the observed variabilities [19]. Even in early studies, CRP and ESR were found to be consistently elevated only in sJIA (systemic juvenile idiopathic arthritis), with uneven results in polyarthritis where they mostly correlate with each other rather than with disease activity, and more recent results demonstrate the dubious value of CRP and ESR as a marker of long-term outcome [21–24]. For the various forms of the proposed disease activity index JADAS, which has been modeled after the DAS28 in RA, ESR or CRP has initially been included as an activity marker [13, 25]. However, the correlation of ESR to the other parameters of the JADAS have been described as moderate at best – slightly better in the case of sJIA – and exclusion of laboratory parameters in the cJADAS has been proven beneficial, not only improving feasibility in the clinical setting but also maintaining sensitivity and specificity of the index [8, 9, 14]. The JADAS ‘overall did not improve by adding the erythrocyte sedimentation rate [9].’

In this study, we show that in the oligoarthritis categories of JIA, the α2-fraction of serum electrophoresis, especially when corrected by total serum protein, shows a markedly higher correlation to disease activity as measured by cJADAS27, compared to the traditional biomarkers of acute phase reaction. Even more striking, haptoglobin as a component of the α2-fraction demonstrates a significantly higher correlation to cJADAS27 than both CRP and ESR in all three subgroups. The observed differences in the two cohorts can be explained by the larger number of samples in the ConfC and the increased homogeneity by using a lesser number of individuals. Considering pseudo R squared of the model, α2-fraction and haptoglobin also represent a higher proportion of the variability than ESR or CRP, with the proportion of the variability explained by haptoglobin as a predictor being three times as high than in CRP. The ROC analysis demonstrated a better utility as a biomarker for both haptoglobin and CA2F, compared to ESR and especially CRP. Using the traditional ranking system for the AUC in the ROC (0.9 – 1.0 = excellent, 0.8 – ≤ 0.9 = good, 0.7 – ≤ 0.8 = fair, 0.6 – ≤ 0.7 = poor, 0.5 – ≤ 0.6 = fail), only haptoglobin falls under the ‘fair’ category, while all other parameters can be considered ‘poor’.

Protein electrophoresis, especially in the more modern form of capillary serum electrophoresis, provides information about the status of protein components that are present in large quantities in the body [26]. It is an inexpensive test, and widely available in hospitals by virtue of its common use to monitor patients with multiple myeloma. The alpha-fractions are considered a typical, if unspecific, biomarker for the acute-phase reaction [12]. Children and elderly people can show higher levels of α2 -macroglobulin and may therefore demonstrate a slightly more pronounced α2-fraction [27]. While immunoglobulins and beta globulins have been studied in juvenile arthritis, alterations in the alpha-fractions have not yet been investigated [28].

Haptoglobin is an acute phase protein which binds free hemoglobin and has anti-inflammatory properties, but may have proinflammatory effects on the joint; its synthesis is induced by various cytokines including IL-1 and IL-6 [29]. It appears to play a role in the inflammatory process of bone destruction via bradykinin and thrombin stimulation of prostaglandin E2 formation, leading to bone resorption [30]. It has also been shown that haptoglobin modulates Th1 versus Th2 balance in macrophages by promoting a Th1 cellular response according to genotype [31].

Haptoglobin has been demonstrated to be a normal constituent of synovial fluid, with a hemoglobin-binding capacity of approximately 40% of that in serum [32]. Almost 40 years ago, it has been demonstrated that for RA, CRP shows significantly higher correlation with disease activity than haptoglobin, and haptoglobin has generally been disregarded as a possible biomarker for rheumatology ever since [33]. In addition, when studying the genetic subtypes of haptoglobin in the synovial fluid in patients with rheumatoid arthritis, no obvious correlation was found with elevated levels in all three haptoglobin types studied [34].

Still, the relationship of haptoglobin to inflammation is well described, with haptoglobin mRNA found to be expressed locally in the joints of arthritic rats [35]. Haptoglobin has also been shown to restore homeostasis after inflammatory processes, and to bind to activated macrophages, therefore inhibiting TNFα production [36, 37]. In fact, elevation of the two α-chains of haptoglobin has been correlated to response to TNF inhibitors in RA [38].

For JIA, Rosenkranz et al. have examined the synovial fluid proteome of various categories; a comparison between the different subtypes revealed 24 significantly differentially expressed proteins of which haptoglobin was the most prominent differentiator [39]. Levels of haptoglobin were markedly elevated in both systemic JIA and polyarticular JIA, with the former demonstrating the greatest overall expression. They also demonstrated by PCR that haptoglobin is produced locally in the inflamed joint, similar to mice [35]. Gibson et al., who demonstrated differential proteome profiles in synovial fluid of untreated polyarticular, persistent and extended oligoarticular JIA patients, showed similar results [40]. Here, they described increases of haptoglobin in all groups, with 2.3- to 4.3-fold overexpression in polyarticular patients when contrasted with the whole group of oligoarticular patients. These findings indicate that elevation of haptoglobin appears to be the direct result of local inflammation in the joint. CRP represents an acute phase protein following IL-6 secretion by macrophages and T cells, and IL-6 has been found in elevated levels in the synovial fluid, but it is produced mainly in the liver [41]. ESR represents the change in the net negative charge of red blood cells, causing them to fall more rapidly in suspension in the presence of acute phase reactants [42]. It has been shown previously, and in this study, to correlate more closely with disease activity and flares in JIA than CRP [43]. However, it is also affected by a large variety of factors beyond the acute phase reaction. A biomarker such as haptoglobin offers the chance of not only accurately depicting the acute phase reaction but also to represent a biomarker originating from the local site of inflammation.

A variety of other conditions may also increase the alpha2-fraction of serum electrophoresis, including corticosteroid therapy and adrenal insufficiency, and levels of haptoglobin, most notably hemolytic anemia. Haptoglobin has also been described as elevated in inflammatory, non-immune mediated disease, most notably osteoarthritis and Perthes disease, and was found locally expressed in oncological tissue [35, 44, 45]. Further studies will have to closely examine the specificity of this biomarker. Disease duration as well as differences in medication are also possible confounding factors and may alter the profiles of inflammatory proteins. In this analysis, fractions of serum electrophoresis were not corrected for age; however, all patients were above the age of 1 year where distribution of the different fractions of electrophoresis show only minimal changes [12]. Interestingly, in this study correlation between all biomarkers, not only haptoglobin, were lower in the extended oligoarthritis category with a longer disease duration, pointing at a possibly limited utility of laboratory biomarkers in JIA in the long term.

## Conclusion

The α2-fraction, corrected for total serum protein, as well as haptoglobin are well established laboratory parameters that can be determined by most laboratories at minimal cost. This study demonstrates that while not perfect, these parameters could well be used as potential biomarkers for joint and disease activity in the most frequent subgroups of JIA, improving both diagnostic accuracy and allowing more accurate tracking of disease activity by laboratory studies, similar to CRP in adult patients. Further studies investigating the value of haptoglobin versus CRP and ESR do present as obvious choices: not only the investigation of the value of haptoglobin in enthesitis-related arthritis and seropositive polyarthritis comes to mind, where a more dominant role of CRP could possibly be expected—similar to adult patients. One could also speculate about the role of haptoglobin in systemic arthritis, where it would not be surprising to correlate with joint activity, while CRP on the other hand possibly correlates with systemic manifestations of the disease. Furthermore, it remains to be seen if addition of haptoglobin to the JADAS improves its value as an activity marker beyond that seen with ESR or CRP.

## Supplementary Information

**Additional file 1: Supplemental Table 1.** Correlation to cJADAS27 – Exploratory Cohort (ExpC).

**Additional file 2: Supplemental Table 2.** Correlation to cJADAS27 – Confirmatory Cohort (ConfC).

**Additional file 3: Supplemental Table 3. **Correlation to Total Active Joint Count and cJADAS27 – Combined Cohorts (ExpC + ConfC).

## Data Availability

The datasets during and/or analyzed during the current study available from the corresponding author on reasonable request.

## Abbreviations

AUC: Area under the Curve
CRP: C-reactive Protein
CA2F: Calculated α2-Fraction of Serum Electrophoresis
cJADAS: Clinical Juvenile Arthritis Disease Activity Score
CondC: Confirmatory Cohort
DAS28: Disease Activity Score 28
ESR: Erythrocyte Sedimentation Rate
ExpC: Exploratory Cohort
IL: Interleukin
ILAR: International League of Associations for Rheumatology
JADAS: Juvenile Arthritis Disease Activity Score
JIA: Juvenile Idiopathic Arthritis; RA: Rheumatoid Arthritis
ROC: Receiver Operating Characteristic
sJIA: Systemic juvenile idiopathic arthritis
TNFα: Tumor Necrosis Factor α
