# Supplementary material for: α2-fraction and haptoglobin as biomarkers for disease activity in oligo- and polyarticular juvenile idiopathic arthritis
Source: Pediatr Rheumatol Online J. 2022 Aug 13;20:66. doi: 10.1186/s12969-022-00721-7 (PMC9375368; doi:10.1186/s12969-022-00721-7)
Supplement: Supplementary file 1 — Additional file 1: Supplemental Table 1. Correlation to cJADAS27 – Exploratory Cohort (ExpC). [file 12969_2022_721_MOESM1_ESM.docx]

**Supplemental Table 1 Correlation to cJADAS27 – Exploratory Cohort (ExpC)**

|  | ESR | CRP | α_2_-Fraction | α_2_-Fraction, calculated | α_2_-Macroglobulin | Haptoglobin | Ceruloplasmin |
| --- | --- | --- | --- | --- | --- | --- | --- |
| Complete sample | 0.270^**^ | 0.181^*^ | 0.226^**^ | 0.238^**^ | -0.181^*^ | 0.325^**^ | 0.266^**^ |
| Oligoarthritis, persistent and extended | 0.370^**^ | 0.223^*^ | 0.345^**^ | 0.354^**^ | -0.135 | 0.400^**^ | 0.378^**^ |
| Oligoarthritis, persistent | 0.354^**^ | 0.175 | 0.400^**^ | 0.402^**^ | -0.238* | 0.468^**^ | 0.417^**^ |
| Oligoarthritis, extended | 0.387^*^ | 0.341 | 0.283 | 0.300 | 0.032 | 0.297 | 0.366^*^ |
| RF-negative Polyarthritis | 0.272^*^ | 0.317^**^ | 0.186 | 0.184 | -0.245^*^ | 0.403^**^ | 0.212 |

* p<0.05, ** p<0.01
