# Supplementary material for: α2-fraction and haptoglobin as biomarkers for disease activity in oligo- and polyarticular juvenile idiopathic arthritis
Source: Pediatr Rheumatol Online J. 2022 Aug 13;20:66. doi: 10.1186/s12969-022-00721-7 (PMC9375368; doi:10.1186/s12969-022-00721-7)
Supplement: Supplementary file 2 — Additional file 2: Supplemental Table 2. Correlation to cJADAS27 – Confirmatory Cohort (ConfC). [file 12969_2022_721_MOESM2_ESM.docx]

**Supplemental Table 2 Correlation to cJADAS27 – Confirmatory Cohort (ConfC)**

|  | ESR | CRP | α_2_-Fraction | α_2_-Fraction, calculated | α_2_-Macroglobulin | Haptoglobin | Ceruloplasmin |
| --- | --- | --- | --- | --- | --- | --- | --- |
| Complete sample | 0.420^**^ | 0.292*^*^ | 0.404^**^ | 0.373^**^ | -0.061^*^ | 0.470^**^ | 0.370^**^ |
| Oligoarthritis, persistent and extended | 0.381^**^ | 0.193^*^ | 0.421^**^ | 0.344^**^ | -0.013 | 0.498^**^ | 0.307^**^ |
| Oligoarthritis, persistent | 0.270 | 0.083 | 0.472^**^ | 0.483^**^ | 0.058 | 0.544^**^ | 0.497^**^ |
| Oligoarthritis, extended | 0.646^**^ | 0.496^**^ | 0.517^**^ | 0.340^**^ | 0.001 | 0.559^**^ | 0.340^**^ |
| RF-negative Polyarthritis | 0.438^*^ | 0.406^**^ | 0.431^**^ | 0.430^**^ | -0.077 | 0.472^**^ | 0.451 |

* p<0.05, ** p<0.01
