# Supplementary material for: α2-fraction and haptoglobin as biomarkers for disease activity in oligo- and polyarticular juvenile idiopathic arthritis
Source: Pediatr Rheumatol Online J. 2022 Aug 13;20:66. doi: 10.1186/s12969-022-00721-7 (PMC9375368; doi:10.1186/s12969-022-00721-7)
Supplement: Supplementary file 3 — Additional file 3: Supplemental Table 3. Correlation to Total Active Joint Count and cJADAS27 – Combined Cohorts (ExpC + ConfC). [file 12969_2022_721_MOESM3_ESM.docx]

**Supplemental Table 3 Correlation to Total Active Joint Count and cJADAS27 – Combined Cohorts (ExpC + ConfC)**

|  | ESR | | | CRP | | | α_2_-Fraction | | α_2_-Fraction, calculated | α_2_-Macroglobulin | | | | Haptoglobin | | | Ceruloplasmin |
| --- | --- | --- | --- | --- | --- | --- | --- | --- | --- | --- | --- | --- | --- | --- | --- | --- | --- |
| Total Active Joint Count | 0.141^**^ | 0.165^**^ | | | 0.077 | | | 0.087 | | | -0.149^**^ | | 0.189^**^ | | | 0.125^**^ | |
| cJADAS27 | 0.318^**^ | | 0.261^**^ | | | 0.286^**^ | | | 0.310^**^ | | | -0.071 | | | 0.413^**^ | | 0.311^**^ |

* p<0.05, ** p<0.01
